# Social housing enhances acquisition of task set independently of environmental enrichment: A longitudinal study in the Barnes maze

**DOI:** 10.3758/s13420-020-00418-5

**Published:** 2020-09

**Authors:** Victoria R. Heimer-McGinn, Taylor B. Wise, Brittany M. Hemmer, Judith N.T. Dayaw, Victoria L. Templer

**Affiliations:** 1 Department of Psychology, Providence College, 1 Cunningham Square, Providence, RI 02918, USA.; 2 Department of Psychology, Roger Williams University, 1 Old Ferry Road, Bristol, RI, 02809, USA

**Keywords:** sociability, acquisition, cognitive flexibility, spatial learning, spatial memory, enrichment

## Abstract

Human studies suggest that healthy social relationships benefit cognition, yet little is known about the underlying neural mechanisms of this protective effect. In rodents, studies on acute isolation and environmental enrichment (EE) confirm the importance of social exposure. Despite the widely recognized importance of sociality, however, rodent models have yet to explore the independent contributions of social housing divorced of other forms of enrichment. This study dissociates the effects of social and physical enrichment on spatial learning and memory from adulthood to old age. Rats were placed in either single or group housing, provided with ample enrichment, and tested at three time-points on several phases/versions of the Barnes maze (BM) (standard, retention probes, variable location, and reversal). We found that sustained social housing enhanced cognitive flexibility, as evidenced by superior acquisition of task set (standard BM), adaptability to a new task set (variable BM), and improved reversal learning (reversal BM). Long-term retention (BM retention probes) of spatial memory was unaffected by housing conditions. Recent studies from our lab, including this report, are the first to show that social housing confers cognitive benefits beyond those of physical enrichment. Importantly, our experimental design is ideal for exploring the neural underpinnings of this socially-induced cognitive protection. Understanding how sociality influences cognition will be invaluable to translational models of aging, neuropsychiatric disease, and neurological injury.

## Introduction

Living in social groups and having healthy social interactions has long been known to have positive effects on cognition, including enhancements in both executive function and memory. The cognitive benefits of sociality have been mostly studied in children and older adults (>65), (Barnes, Mendes de Leon, Wilson, Bienias, & Evans, 2004; Berkman, 2000; Hitchcock, Ellis, Williamson, & Nixon, 2015; Holtzman et al., 2004; Howrey, Raji, Masel, & Peek, 2015; Huxhold, Miche, & Schuz, 2014; Kang, Boss, & Clowtis, 2016; Kats et al., 2016; Krause, 2007; McQuade, Murray-Close, Shoulberg, & Hoza, 2013; Zuelsdorff et al., 2017), and in clinical recovery settings, (Amieva et al., 2010; Gallagher et al., 2016; Milan, Zona, Acker, & Turcios-Cotto, 2013), while a handful of studies also extend these findings to healthy adults (ages 30–65) (Karlamangla et al., 2014; Seeman et al., 2011). Despite the importance of sociality in the human literature, however, very little is known about the neural mechanisms that underlie the cognitive benefits of social enrichment. This is because rodent models, which allow for tighter controls and more extensive experimental manipulation, have not yet been used to explore the independent contributions of sociality in a way that is relevant to the human experience.

Like humans, rodents live in complex social structures, are able to perform a wide variety of cognitive tasks (Morellini, 2013), and experience severe cognitive deficits when raised in acute isolation, which is defined by absence of enrichment, cage mates, or human handling (Fone & Porkess, 2008). Accordingly, social housing has become one of the key components of laboratory environmental enrichment (EE) in rodent work (Simpson & Kelly, 2011). Understanding the deleterious effects of acute isolation and the benefits of EE has dramatically changed standard laboratory practices and influenced our understanding of animal welfare. However, we still lack animal models that explore the cognitive benefits of social enrichment alone for subjects that are otherwise healthy and/or enriched. Such a design – where housing is non-social but not deprived or entirely isolated – would yield results that are relevant to the general human population and not limited to people raised in extreme social isolation (e.g. agoraphobia). Thus, an experimental design applying social housing as an independent variable, with other forms of enrichment held constant, is necessary to clarify how sociality independently influences cognition in humans.

Furthermore, it is important to point out *why* decades of work on the cognitive benefits of EE (reviewed in Fischer, 2016; Frick & Benoit, 2010) have been unable to dissociate the effects of social and physical enrichment. The most obvious reason is that EE generally includes both physical (e.g. toys, running wheels) and social (i.e. number of rats per cage, handling) enrichment (Simpson & Kelly, 2011), so direct assessment of the contributions of each is not feasible. The second, and more nuanced reason is that the control condition is not consistent across studies (Simpson & Kelly, 2011). In some studies, the control group is socially isolated (Moritz, Geeck, Underly, Searles, & Smith, 2014), meaning that any benefits observed can be attributed to the combined effects of social and physical enrichment. In other studies, both the control and EE groups are socially housed, with equal number of subjects per cage (e.g. 4–5 in Gortz et al., 2008; Reichmann et al., 2016), so that any benefits observed cannot be attributed to social enrichment. In yet another group of studies, the control group is housed in “standard laboratory conditions” (i.e. 2–5 per cage) so that both groups are “socially” housed but the EE group has more subjects per cage (e.g. 10 vs 2 compared to 8 vs 5) (Bonaccorsi et al., 2013; Harati et al., 2011; Harburger, Lambert, & Frick, 2007; Harburger, Nzerem, & Frick, 2007; Madhavadas, Subramanian, & Kutty, 2017). Although this last category of studies has been instrumental in highlighting the benefits of EE over standard housing conditions, they tell us nothing about the individual effects of sociality. If sociality does, in fact, provide benefits beyond those of physical enrichment, we would expect to see inconsistencies across studies depending on the number of subjects per cage in the control group. This is indeed what the literature shows as described below.

Parsing out the inconsistencies in the literature by social housing conditions can reveal insightful trends. One area of EE literature where inconsistent results could indicate that social enrichment confers unique cognitive benefits, is spatial learning in healthy adults. Although EE (compared to either social or nonsocial controls) consistently improves spatial learning and memory in compromised subjects, including models of aging, disease, injury, or experimental lesions, the same is not always true for the healthy adult controls in these studies. For instance, EE is often found to enhance spatial learning and memory in the experimental subjects but not in the control group (e.g. wildtype, sham, adult) (Gortz et al., 2008; Harburger, Lambert, et al., 2007; Harburger, Nzerem, et al., 2007; Jacotte-Simancas, Costa-Miserachs, Torras-Garcia, Coll-Andreu, & Portell-Cortés, 2013; Madhavadas et al., 2017; Peruzzaro et al., 2013; Reichmann et al., 2016). The fact that EE provided no benefits to healthy adults is rarely even mentioned in these studies, let alone discussed. In contrast, three studies do find enhancements to spatial learning in healthy adult controls (Bonaccorsi et al., 2013; Harati et al., 2011; Moritz et al., 2014). One common thread among these last three studies is that the EE and control groups had vastly different numbers of subjects per cage, meaning either social or physical enrichment, or both, could have been responsible for the cognitive enhancements. Taking this into consideration, it is likely that conflicting results in other studies arise because social housing was not consistently controlled within or across studies. Thus, it is possible that in healthy adults it is actually the social, and not the physical aspects of EE, which are responsible for spatial learning enhancements.

The purpose of the current study was to assess the influence of social housing on spatial learning and memory in equally enriched physical environments. Our studies (Templer, Wise, Dayaw, & Dayaw, 2018b; Templer, Wise, & Heimer-McGinn, 2018) are the first to use a model in which social housing is an independent variable so that the potential effects of sociality are assessed beyond those of practice, exercise, and physical enrichment. Adult rats were placed in life-long social (SH) or nonsocial housing (NSH) and tested on several versions of the Barnes maze at three points in their lifetime; adult (5 months), middle age (14 months), and old age (22 months). Both groups were placed in the same colony room in large, multi-level cages, handled extensively, and provided with ample physical enrichment and access to self-directed exercise. NSH subjects were individually housed but not strictly “isolated” because they were handled extensively and could smell and view rats in other cages. This means that the translational relevance of our results can extend to people with minimal social interactions, rather than being limited to people who live in extreme isolation with no physical enrichment. BM testing included standard training with consistent goal box (GB) location, retention probes, variable location training, and reversal learning. We hypothesized that social housing would confer cognitive benefits beyond those of the physical components of EE throughout the lifespan. This was based on the knowledge that EE, which included social housing, benefited spatial learning in middle to old age in most previous studies, and in adults only when EE controls were nonsocially housed. Because both of our groups were enriched in every sense except the social aspect, our results are potentially relevant to the general human population and not only to those who lead extremely sedentary lifestyles.

## Method

**Figure 1.**
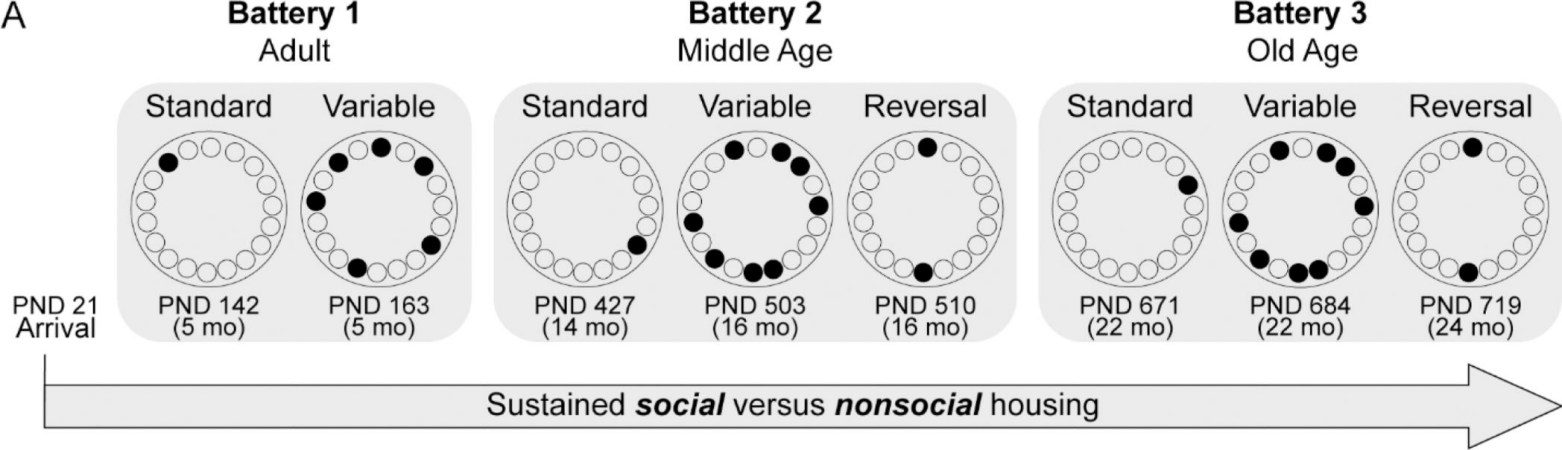
Experimental design and timeline. Rats were maintained in either social or nonsocial housing conditions from arrival (post-weaning) to old age. Three versions of the Barnes maze (BM), standard BM, variable BM, and reversal BM, were conducted within each of the three testing batteries as depicted. Batteries 1, 2, and 3 were administered during adulthood, middle age, and old age, respectively. Correct goal box (GB) locations, where cereal treats could be found, are indicated as filled (black) circles. In the variable BM, the GB changed at every trial, all of which are shown simultaneously. For more detailed information, see Table 1.

We tested whether sustained social (SH) versus nonsocial housing (NSH) influenced cognitive function. Rats were tested on three repetitions of a behavioral battery (B1, B2, and B3), each of which was performed at a different life stage; late adulthood, middle age, and old age (5, 14, and 22 months respectively; Figure 1). Each subject was tested on various versions of the BM. It is important to note that because different versions of the task were presented repeatedly, task demands were constantly changing and cognitive flexibility was required to successfully switch between task sets. Therefore, only the first repetition of the standard task assessed spatial learning and long-term retention of spatial memory. Thereafter, the changing task demands tested the ability to successfully switch between task sets. A simple discrimination task was used to assess learning in a non-spatial context and these results can be found in the Supplemental Materials. We refer to *task set acquisition* as a cognitive approach to the initial “rule” learning of a task. *Task set* thus includes aspects of the BM task that are consistent across trials and must be learned to perform well in the task. Sometimes, especially in the nonhuman animal literature, these “rules of the game” are referred to as reference memory (Shettleworth, 2010).

### Subjects

Twenty adult male Long Evans rats were used in this long-term study (Charles River Laboratories, Boston, MA, USA). Upon arrival, rats were randomly assigned and separated into one of two groups: 10 rats were socially housed (SH) in one cage and 10 were nonsocially housed (NSH) in individual cages. One rat in the social group died of natural causes partway through the experiment and was therefore excluded from the analyses. All rats were born on the same day from separate litters and arrived at post-natal day (PND) 21. Rats were maintained at 21.6 °C and kept on a 12:12 reversed light-dark cycle with *ad libitum* access to food and water. Five weeks before behavioral testing, rats were food-restricted and maintained at 85–90% body weight. All testing occurred during the dark phase of the light cycle, at approximately the same time each day. Rats were 5, 14, and 22 months old (PND 143, 428, and 672 respectively) at the start of each behavioral battery. Aside from the tasks described in this paper, these rats were subjected to the radial arm maze, open field observations, (Templer, Wise, & Heimer-McGinn, 2018), the social interaction paradigm (Templer, Wise, Dayaw, & Dayaw, 2018a) and a continuous T-maze task with a differential reward (Hemmer et al., 2019). The results of this overall longitudinal study were published in separate reports to allow us to delve deeper into each task. These experiments were carried out in accordance with NIH guidelines for the care and use of rats in research. All procedures described in the current study were approved by the Institutional Animal Care and Use Committee (IACUC) of Providence College.

### Housing

Upon arrival, rats were immediately assigned to one of two groups randomly; socially housed (SH, n=9) or non-socially housed (NSH, n=10). SH rats had continual access to their cage mates, whereas NSH rats could smell, hear, and see others in the room but were physically isolated. Aside from social grouping and overall cage size, both groups lived in identical conditions. For several weeks upon arrival rats were kept in plastic shoebox cages, NSH rats individually and SH rats in two boxes of five, until they were large enough to be housed permanently in enriched housing. Permanent housing consisted of large open cages enclosed by metal bars, with multiple platforms, corncob bedding, and a tray at the bottom (Ferret Nation). All nine SH rats were housed together in a cage measuring 36 × 24 × 63 in, and each NSH rat was housed in a smaller cage measuring 17 × 12.75 × 24 in. Cage sizes were designed according to a density analysis: SH cage = 5,443 in^2^ per animal (54,432 in^2^ total) and NSH cage = 5,202 in^2^ per animal. Controlling the rat-to-cage size ratio was necessary in order to prevent over-crowding and equalize net space for physical activity across groups, thereby eliminating potentially confounding variables. Importantly, both cage sizes were large enough to accommodate ample and identical environmental enrichment, including platforms, running wheels, plastic toys/enclosures, an open-topped plastic shoebox cage (17 × 8 × 8 in) and wooden chew toys. Access to physical activity was therefore identical for both groups. To assist with identification, all rats were periodically dyed with non-toxic pet hair dye gel (Top Performance).

### Apparatus

#### Barnes maze tasks.

Testing was performed on a white circular acrylic platform (48 inch diameter) with 18 equally spaced holes (3.75 inch diameter) located 1.5 inches from the edge, and no surrounding walls (Noldus Information Technologies, Leesburg, VA, USA). The maze was elevated 52 inches from the floor and rats were transported from their home cage to the behavioral platform in a start chamber (10.5 in diameter x 5 in height) with an opaque domed lid. A black goal box (GB; 15.5 × 5 × 3.25 in) could be secured under any of the holes in such a way that rats could not visibly discriminate its location from other holes. Except where indicated, the GB was baited with a piece of cereal (Kellog’s Fruit Loops, USA). Additional pieces of cereal were attached at the side of each hole to avoid obvious olfactory cues; rats could neither see nor reach any of these pieces. Other than standard room lights, which facilitated video capture, no aversive motivators were used. Between trials, the maze and GB were wiped clean with 70% isopropyl alcohol solution and allowed to dry. The apparatus was housed in a dedicated testing room (11.5 × 18 ft.) that contained several environmental cues that were consistent for the entirety of an experiment. These included a computer desk, chairs, whiteboards, and a table. The position of the experimenter, who remained still and quiet, was also kept constant.

### Behavioral Procedure

#### Standard Barnes maze (BM) task.

The training phase consisted of 8 trials performed across 4 days, with 2 trials per day, and the location of the GB remaining constant through the task. The task was repeated across three behavioral batteries, which we called battery 1 (B1), battery 2 (B2), and battery 3 (B3). For each battery, a different hole held the baited GB. See Figure 1 and Table 1 for an experimental timeline, and specific postnatal days. Before each trial, rats were placed in the center of the maze inside the start chamber. Trials began when the domed lid was removed from the start chamber, and the rat was free to explore. Trials ended after 180 sec or when the rat entered the GB, whichever came first. Each day rats, were tested in the same random order within their groups, and the relative order of the two groups alternated on each testing day. Prior to training in B1, rats were habituated over several days to the maze and the GB. Habituation trials involved retrieving several cereal pieces in the GB; at each successive trial, the number of cereal rewards was decreased and these were placed further towards the back of the box. Several days after the final training session, rats were tested on retention probe trials that were similar to training except without a baited GB. In batteries 1, 2, and 3, rats received 4, 2, and 3 probe trials respectively. In B1 they were done at 8, 9, 10 and 11 days after the last training trial; in B2, 5 and 7 days after; and in B3, 3, 5, and 7 days after.

#### Variable BM task.

The task consisted of 8 trials, each with a new GB location. Each GB location was at least 4 holes away from the preceding and proceeding baited locations. Running conditions and protocols were the same as for the standard BM. This task was run in all 3 batteries (B1, B2 and B3).

#### Reversal BM task.

The task consisted of 8 trials with the GB in one consistent location (standard phase; Std) followed by 8 trials with the GB in a new consistent location (reversal phase; Rev). The new locations were always at least 4 holes away from the original. Running conditions and protocols were the same as for the standard BM. This task was only run in B2 and B3.

### Data Analysis

#### Barnes maze tasks.

Videos were captured and tracked using Ethovision XT 9 (Noldus Information Technology, Inc., Leesburg, VA). Primary latency, the time it takes the rat to find the GB, even if they do not enter immediately (Harrison, Reiserer, Tomarken, & McDonald, 2006), was calculated in Ethovision and verified manually by a blinded experimenter. Trials to criterion (TTC) was defined as the number of trials it took the rat to reach the GB in under 6 sec. Perseverative errors, the number of nose pokes in a hole that previously contained the GB, were analyzed for the first three trials of the variable and reversal BM tasks. Search strategies were classified as “direct” (going straight to the GB), “serial” (going around the perimeter of the maze in clockwise or counter-clockwise fashion), or “mixed” (multiple crossings through the center of the maze) (Sunyer, Patil, Hoger, & Lubec, 2007). Errors and search strategies were scored manually by a blinded experimenter using trial videos.

### Statistical Analysis

#### Barnes maze tasks.

Primary latency for each task was analyzed by repeated measures analysis of variance (rANOVA) using ‘battery’ (1–3) and ‘trial’ (1–8) as within-subject factors and ‘group’ (non-social, social) as the between-subject factor. In addition, planned analyses of each battery were performed using ‘trial’ and ‘group’ as within- and between-subject factors respectively. Learning curve slopes were analyzed using linear regression with ‘trial’ as the independent variable and ‘latency’ as the dependent variable. TTC was analyzed by rANOVA using ‘battery’ and ‘group’ as within- and between-subject factors respectively, as well as by planned one-way ANOVA comparing the two housing groups within each battery. Search strategies were analyzed by rANOVA using ‘battery’ and ‘strategy’ as within-subject factors and ‘group’ as the between-subject factor. Significant interactions were further investigated using rANOVA for each battery and each strategy. Where overall significance was found, housing groups were compared to each other using one-way ANOVAs. Perseverative errors were averaged across the first three trials for each housing group and analyzed by rANOVA using ‘battery’ and ‘group’ as within- and between-subject factors respectively. Sphericity was assessed by Maulchy’s test; when significant, the Greenhouse-Geisser correction was applied. All analyses were performed in SPSS Version 24 (IBM, USA).

## Results

### Standard BM

**Figure 2.**
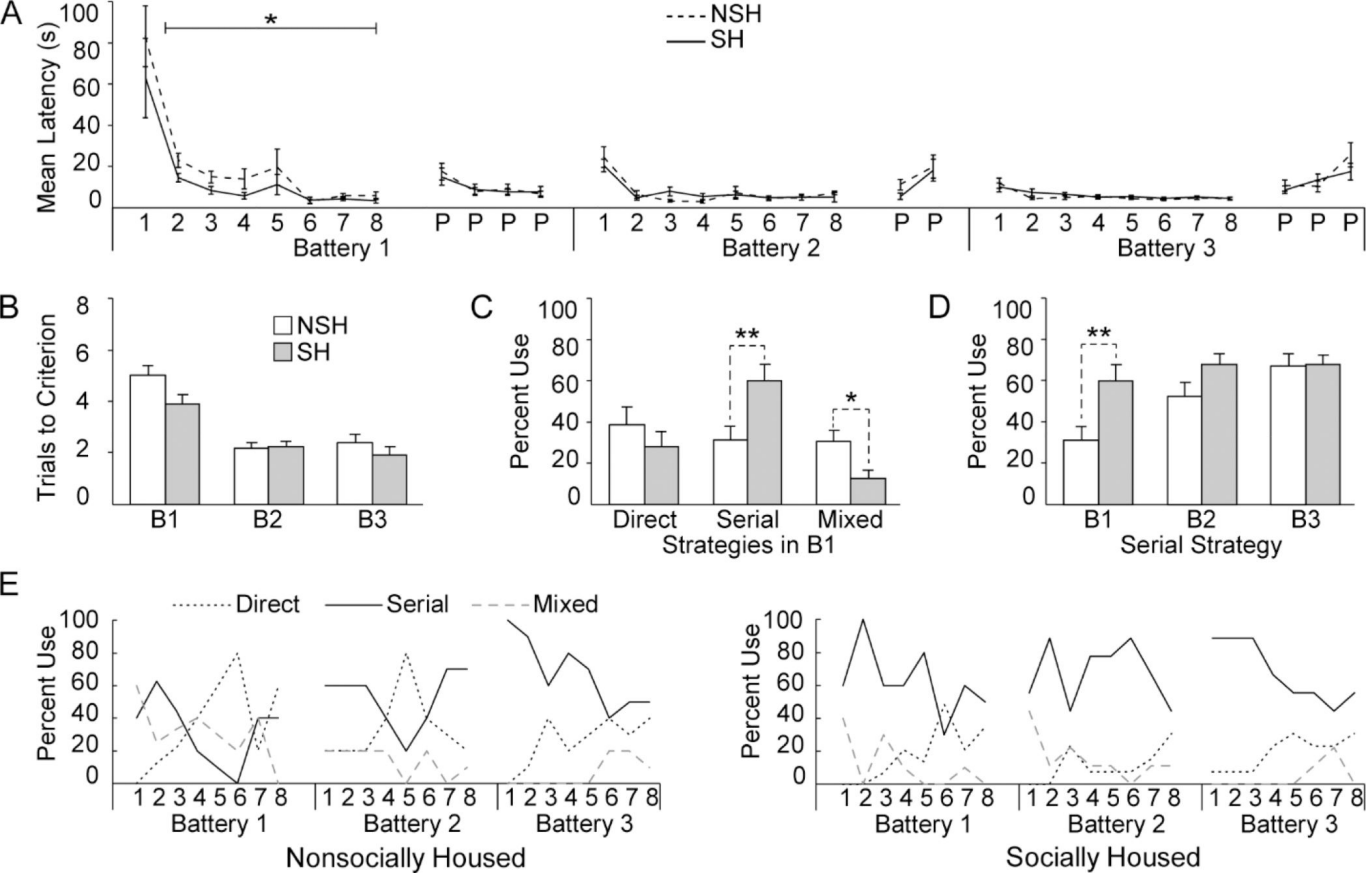
Performance on standard Barnes maze. The goal box (GB) remained constant throughout each battery but changed across batteries. **A)** Mean primary latency to reach the GB during training trials (1–8) and during retention probes (P) in Batteries 1, 2, and 3 (B1, B2, and B3, respectively). Probes were performed 8, 9, 10 and 11 days after the last training trial in B1; 5 and 7 days after in B2; and 3, 5, and 7 days after in B3. In the training phase of B1, nonsocially housed (NSH) rats took significantly longer than socially housed (SH) rats to find the baited GB (‘group’ effect F_1,18_=5.067, *p*=0.037). No other significant differences were observed. **B)** Mean trials to criterion in B1, B2, and B3 were defined as the number of trials it took to reach the GB in under six seconds. In B1, there was no significant difference, although there was a trend for SH rats to require less trials to reach criterion compared to NSH rats (F_1,19_=4.048, *p*=0.059). **C)** Mean percent use of strategies in B1. The SH group used the serial strategy more than the NSH group (F_1,19_=9.449, *p*=0.007) and the NSH group used the mixed strategy more than the SH group (F_1,19_=8.007, *p*=0.011). **D)** Mean percent use of the serial strategy across batteries. While the NSH group gradually adopted a preference for the serial strategy across batteries, the SH group employed it throughout all three batteries. See Figure S1-S2 for all batteries. Error bars represent standard error of the mean. Significance: * indicates *p*<0.05, ** indicates *p*<0.01. E) Use of strategies across trials. For each trial, data points represent the percent of subjects per group that used a given strategy. The left panel depicts the nonsocial group while panels on the right side depict the social group.

In each of the three batteries, rats performed 8 trials in which the GB remained constant. A new GB location was used for each battery. In the training phases, SH rats performed better than NSH rats in B1 but not in B2 or B3, as evidenced by significantly lower latencies to reach the GB (Figure 2A) and a tendency towards lower trials to criterion (TTC; Figure 2B). rANOVA of primary latency showed that the SH group had a shorter primary latency compared to the NSH group, as confirmed by a main effect of ‘group’ (F_1_,_18_=5.067, *p*=0.037, η^2^ =0.237) in B1, but not in B2 (*p*=0.934) or B3 (*p*=0.433), further indicating group differences in the training phase of testing. Although the effect size was small, the results were nonetheless significant. In all three batteries, there were significant effects of ‘trial’ (B1: F_1 5_,_26_._7_=21.745, *p*=0.000, B2: F_2_._7_,_47_._1_=17.865,*p*=0.000, B3: F_2_._7_,_45_._9_=7.875,*p*=0.000) but not ‘trial x group’ (*p*=0.587 for B1; *p*=0.461 for B2; *p*=0.422 for B3), which shows that performance consistently improved across trials. Similarly, the SH group tended to have lower TTC compared to NSH (Figure 2B) in B1. This is evidenced by a marginal ‘group’ effect in B1 (F_1_,_19_=4.048, *p*=0.059) but not in B2 (*p*=0.941) or B3 (*p*=0.285). In B1, group differences in performance could not be explained by a group difference in latency to start exploring (*p*=0.248), which would have been evidenced had there been a difference in anxiety related behaviors (e.g. freezing). In addition, there were no group differences in long-term retention of spatial memory as indicated by the retention probes (Figure 1A; B1: *p*=0.995; B2: *p*=0.429; B3: *p*=0.499). Probes were performed 8, 9, 10 and 11 days after the last training trial in B1. Shorter delays were used in B2 (5 and 7 days) and B3 (3,5, and 7 days) to ensure that the lack of group difference replicated at earlier time points. Taken together, these data indicate that SH subjects outperformed NSH housed subjects when first acquiring the task set (i.e. learning the rule that the GB location remained constant) in B1. Consistent with this acquisition benefit hypothesis, cognitive enhancements were not seen in the subsequent training phases (B2, B3) or in the retention probes when the task set had been learned. This indicates that sociality confers cognitive benefits to early learning but not spatial or long-term memory.

While a group difference in training was clearly evident, we wondered if SH versus NSH rats relied on different search strategies to find the correct GB in B1. We indeed observed a ‘strategy x group’ effect in B1 (F_2_,_36_=5.73 2, *p*=0.007) (Figure 2C). The serial strategy was used by the SH more than the NSH group (F_1_,_19_=9.449, *p*=0.007), while the mixed strategy was used by the NSH more than the SH group (F_1_,_19_=8.007, *p*=0.011), and the direct strategy was used equally by both (*p*=0.263). There were no other significant ‘strategy x group’ interactions (Supplemental Figure 1). The use of serial strategy declined for both groups across trials and the use of direct strategy increased across trials, demonstrating spatial acquisition for both groups (Figure 2E). A comparison of strategy uses by group in B1 (Figure 2C) revealed that while the SH group had a preference for strategy (F_2_,_29_=17.695, *p*=0.000), the NSH group did not (*p*=0.663). Post-hoc tests revealed that the SH group preferred the serial strategy over both the direct (*p*=0.001) and mixed (*p*=0.000) strategies. Importantly, percent use of the serial strategy in B1 was strongly and inversely correlated with the decreased latency to GB (r_19_= −0.641, *p*=0.003). Thus, upon first presentation of the standard BM, the social group preferred to explore the arena using the serial strategy while the nonsocial group used all strategies equally, including the inefficient mixed strategy. Taken together, preference for serial strategy early on likely accounted for the enhanced performance of SH compared to NSH rats in B1.

It is important to note that in between the standard BM tasks across batteries, rats were subjected to the variable BM task version, which required rats to learn that the GB location was variable. When rats were presented once again with the standard BM in B2 and B3 they had no way to predict that the task set once again required them to memorize a consistent GB location. In fact, by this point they might have learned that, overall (i.e. across tasks), employing the serial strategy was the most efficient way of maintaining low latencies in light of the changing task rules. We wondered if there was a clear progression in adopting the serial strategy across batteries. We also wondered whether the two housing groups acquired the serial strategy at different rates. For this reason, we examined whether percent use of the serial strategy changed across batteries (Figure 2D). Indeed, we found ‘group’ (F_1_,_17_=9.347, *p*=0.007) and ‘battery’ (F_2_,_34_=6.147, *p*=0.005) effects confirming that use of strategies changed across batteries and that the two groups were different. Follow up one-way ANOVAs showed that the SH group used the serial strategy significantly more often than the NSH group in B1 (F_1_,_19_=9.449, *p*=0.007), but not in B2 (*p*=0.095), or B3 (*p*=0.941). Together, these data indicate that while the NSH group gradually adopted a preference for the serial strategy across batteries, the SH group employed it earliest and throughout all three batteries.

### Variable BM

**Figure 3.**
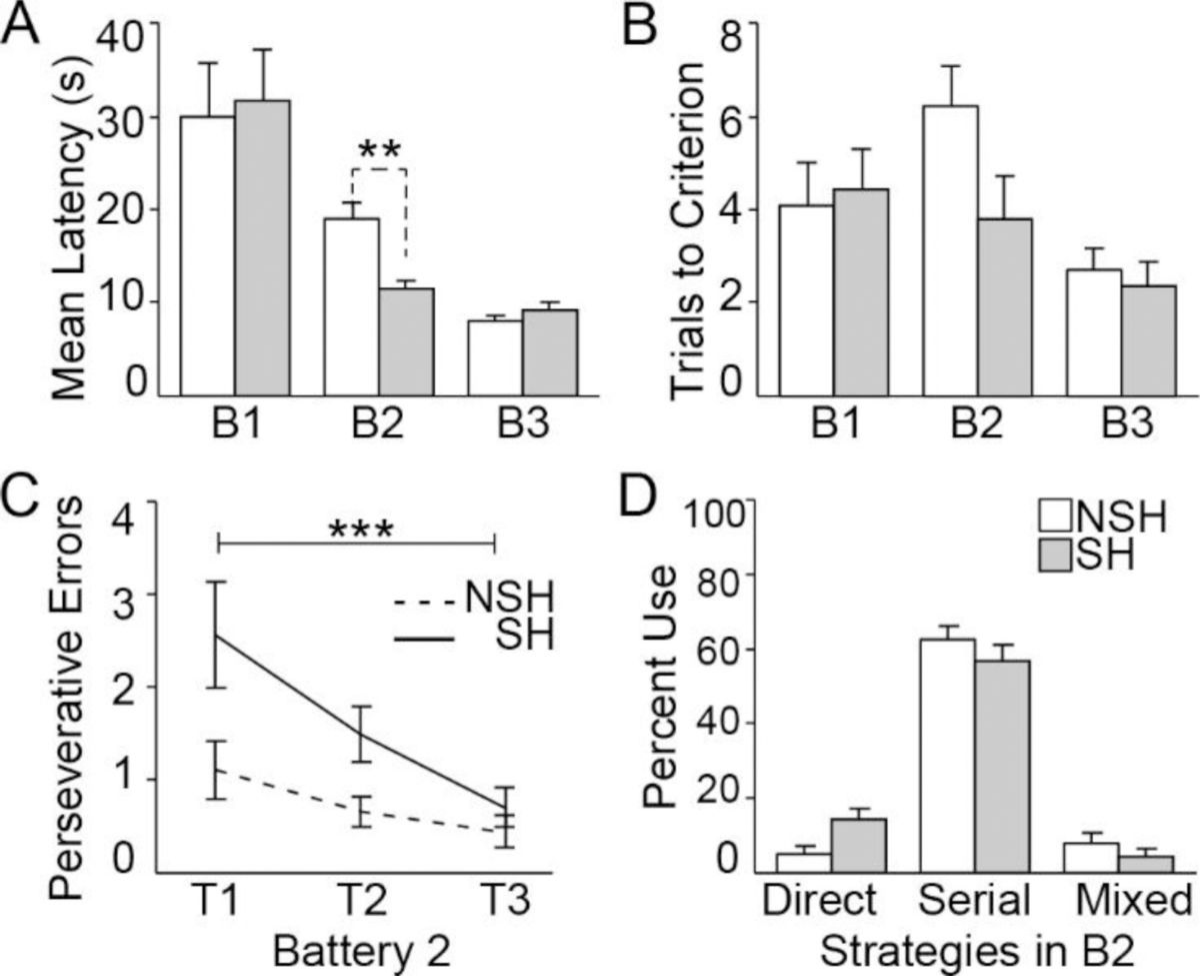
Performance on variable Barnes maze. The location of the goal box (GB) changed on every trial in each battery. **A)** Mean primary latency to reach the GB averaged across trials for batteries 1, 2, and 3 (B1, B2, and B3, respectively). In B2, nonsocially housed (NSH) rats took significantly longer than socially housed (SH) rats to find the baited GB (F_1,17_=12.398, *p*=0.003). No other significant differences were observed. **B)** Mean trials to criterion in B1, B2, and B3 were defined as the number of trials it took to reach the GB in under six seconds. In B2, there was no significant difference, although SH rats tended to require less trials to reach criterion compared to NSH rats (F_1,18_=3.602, *p*=0.075). **C)** Mean perseverative nose pokes into the hole that was baited in the preceding standard task. The NSH group committed more perseverative errors in the first three trials of B2 (T1-T3) compared to the SH group (‘group’ effect F_1,17_=16.139, *p*=0.001). **D)** Mean percent use of strategies in B2. Both groups preferred the serial strategy over the mixed and direct strategies (see Figure S1-S2 for B1 and B3). Error bars represent standard error of the mean. Significance: ** indicates *p*<0.01.

In each of the three batteries, rats performed 8 trials in which the GB location was variable (at a new location for each trial). SH rats performed better than NSH rats in B2 but not in B1 or B3, as evidenced by significantly lower latencies (Figure 3A) and a trend towards lower TTC (Figure 3B). A ‘group’ difference in B2 (F_1_,_17_=12.398, *p*=0.003), indicated that the SH group had significantly lower primary latencies than the NSH, but there were no differences in B1 (*p*=0.846), or in B3 (*p*=0.311). We observed an overall ‘battery’ effect (F_1.1_,_18_._7_=28.340, *p*=0.000), suggesting that rats improved across batteries; pairwise comparisons revealed that all batteries were significantly different to each other. Similarly, the SH group tended to have lower TTC than the NSH group in B2 (F_1.18_=3.602, *p*=0.075) but not in B1 (*p*=0.818) or B3 (*p*=0.610). Taken together these data indicate that the SH group outperformed the NSH group in B2, when rats acquired the new task set, but not in B1 when the task was not yet learned or B3, when the task set was already acquired.

Since the GB location changes at every trial in this task, we wondered whether a difference in perseverative nose pokes (i.e. exploring holes that previously contained the GB) could account for the performance differences in B2. As expected, we observed that the NSH group made more perseverative errors than the SH group (Figure 3C). This was revealed by a ‘group’ effect that was observed in B2 (F_1_,_17_=16.139, *p*=0.001) but not in B1 (*p*=0.781) or B3 (*p*=0.632). Importantly, number of perseverative errors in B2 was positively correlated with latency to GB (r_19_= 0.493, *p*=0.032). A ‘trial’ effect in B2 (F_2_,_34_=6.245, *p*=0.005) indicated that rats made less perseverative errors as trials progressed. Thus, SH rats made less perseverative errors than NSH housed rats, which likely accounted for their decreased latencies to reach the GB.

Finally, we assessed whether search strategies could also account for performance differences in B2, as they had in the standard BM. We found no ‘strategy x group’ interactions across batteries (*p*=0.851) or in B2 (*p*=0.157), indicating that use of strategy did not differ by housing group (Figure 3D). A ‘strategy’ effect (F_2_,_34_=117.248, *p*=0.000) revealed that in B2 both groups equally preferred serial over direct (*p*=0.000) or mixed (*p*=0.000) strategies. Across batteries, both groups progressively acquired the serial strategy (‘battery’: F_2_,_34_=51.314, *p*=0.000). For strategy use across batteries and trials, see Supplemental Figures 1 & 2. In summary, both groups used similar strategies in B2 and across batteries. Thus, enhanced acquisition of task set by SH animals observed in B2 cannot be explained by a cognitive benefit mediated by search strategy use as found in the standard BM.

### Reversal BM

**Figure 4.**
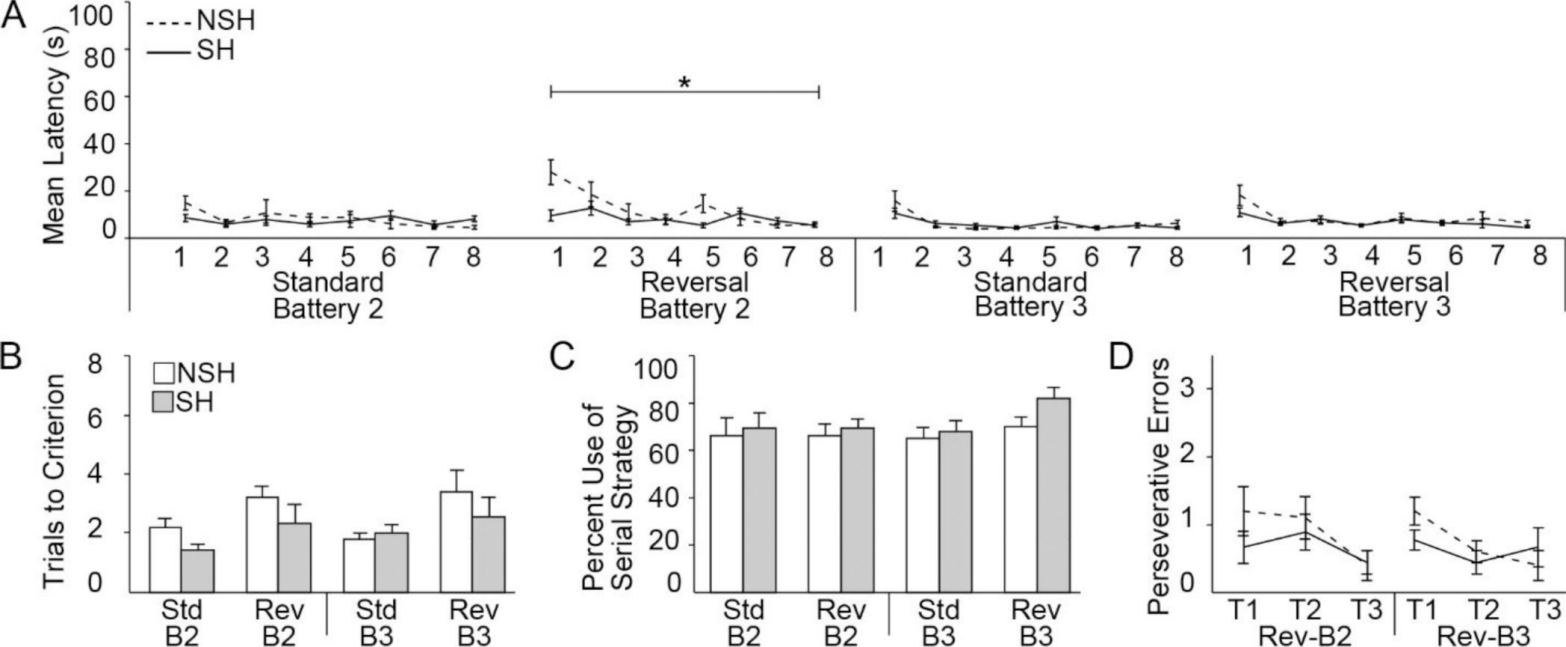
Performance on reversal Barnes maze. For each battery, a standard training phase (Std) was followed by a reversal phase (Rev) in which the location of the goal box (GB) changed. Within each phase, the GB remained constant across trials. **A)** Mean primary latency to reach the GB during across trials (1–8) during standard training and reversal learning in Batteries 2 and 3 (B2 and B3, respectively). The nonsocially housed group (NSH) took significantly longer than the socially housed group (SH) in the reversal phases, as evidenced by a ‘phase x group’ interaction (F_1,17_=8.452, *p*=0.010) but no battery interactions. **B)** Mean trials to criterion in B2 and B3 were defined as the number of trials it took to reach the GB in under six seconds. A main effect of ‘phase’ indicated that both groups took less trials to reach criterion in the standard compared to the reversal phases (F_1,17_=8.410, *p*=0.010), but no ‘group’ or ‘group x phase’ interactions were observed. **C)** Mean percent use of serial strategy in the standard and reversal phases of B2 and B3. No overall differences were observed. Both groups preferred the serial strategy over the mixed and direct strategies in all phases (Figure S1-S2). **D)** Mean perseverative nose pokes into the hole that was baited in the preceding standard phase. During the reversal phases of B2 and B3, both groups committed similar numbers of perseverative errors in the first three trials (T1-T3). Error bars represent standard error of the mean. Significance: * indicates *p*<0.05.

In B2 and B3, rats performed a task that had two phases; 8 trials with the GB in one consistent location (standard phase; Std) followed by 8 trials with the GB in a new consistent location (reversal phase; Rev). We found that SH rats performed slightly better than NSH rats in the reversal phases of B2 and B3, as evidenced by shorter latencies (Figure 4A) and lower, though not significant, TTC (Figure 4B). As expected, an overall main effect of ‘phase’ was observed (F_1_,_17_=26.741, *p*=0.000), indicating that all rats performed better in the standard compared to the reversal phases. An overall interaction of ‘phase x group’ (F_1_,_17_=8.452, *p*=0.010), followed by post-hoc rANOVAs for each phase, revealed that the SH group had significantly lower latencies compared to the NSH group in the reversal phases (F_1_,_17_=4.932, *p*=0.040) but not in the standard phases (*p*=0.632). Furthermore, while the SH group performed consistently well in the reversal phases (see SH flat line in Figure 4B), the NSH group displayed a more typical learning curve. This was confirmed by regression analysis showing a slope of − 1.756 for the NSH group (F_1_,_159_=27.370, *p*=0.000; R^2^=0.148), compared to a slope of −0.569 for the SH group (F_1_,_143_=10.388, *p*=0.002; R^2^=0.068). TTC analyses confirmed a main effect of ‘phase’ (F_1_,_17_=8.410, *p*=0.010), but did not reveal any significant ‘group’ (*p*=0.163) or ‘phase x group’ interactions (*p*=0.419) (Figure 4B). Taken together, these data show that both groups performed better in the standard compared to reversal phase in B2 and B3 but that the SH performed better than the NSH group in the reversal phases.

To determine whether search strategies and/or perseverative errors could account for the performance differences seen in the reversal phase of B2 and B3, we examined use of search strategies. For serial strategy, we found no main effect of ‘group’ (*p*=0.100) or ‘phase’ (*p*=0.959) (Figure 4C). A ‘battery x phase’ interaction (F_1_,_17_=6.991, *p*=0.017) followed by post-hoc tests revealed a ‘phase’ effect in B3 (F_1_,_17_=5.5.24, *p*=0.031) but not in B2 (*p*=0.176), indicating that in B3, both groups used the serial strategy more in the reversal compared to the standard phase. There were no group effects, however. Comparing all strategies, we found no ‘strategy x group’ interaction overall (*p*=0.116), in B2 (*p*=0.263) or in B3 (*p*=0.201), indicating that use of serial, mixed, and direct strategies was similar between groups (Supplemental Figure 1). Similarly, we did not find any group differences in perseverative errors committed by the SH compared to the NSH group in either B2 (*p*=0.268) or B3 (*p*=0.564). In summary, there was no evidence that either search strategies or perseverative errors could account for the group difference in performance during reversal.

## Discussion

In this study we set out to determine how sustained social housing affects flexibility to a constantly changing task set from adulthood to middle and old age. Subjects were placed in single or group housing, provided with ample environmental enrichment and tested on several versions of the BM (standard training, retention probe, variable location, and reversal learning). We found that SH rats exhibited faster acquisition of task set in the standard and variable location versions of the task and slightly faster in the reversal learning task. Long-term retention of spatial memory, as assessed by standard probes, was not affected by social housing at any age. In the standard task, enhanced performance in the SH group was associated with increased use of the serial strategy early on in training. While a direct strategy is indicative of spatial learning, serial exploration of a new environment is in fact the most efficient approach to finding the GB faster when spatial information is not yet available. In the variable location task, SH performance was associated with a decreased number of perseverative errors, indicating that SH rats were not using spatial strategies. Because the constantly changing task demands discouraged spatial learning, once again the procedural strategy employed by SH rats was in fact the most efficient. Overall, our results suggest that social housing improves acquisition of task set and cognitive adaptability, but not spatial learning itself.

### Social housing improves task set acquisition in the standard BM

Group housing in the current study resulted in enhanced acquisition of task set upon first presentation of the standard BM. SH subjects were faster at reaching the GB in the first battery, and this enhanced performance, surprisingly, was strongly correlated with increased use of the serial strategy. Upon closer investigation, it became clear that although both groups displayed spatial learning, as evidenced by increasing use of the direct strategy across trials, the two groups used different exploratory approaches early on. Although the serial strategy is procedural rather than spatial, it is the most efficient way of acquiring spatial information and reaching the GB sooner when first placed in an unfamiliar environment. Indeed, rodents in the BM tend to use the serial strategy more frequently earlier on in training, and then move to more spatial strategies as they commit the GB location to memory (Harrison et al., 2006; Illouz et al., 2016; O’Leary & Brown, 2013). Across batteries, both groups gradually adopted this preference, which likely accounted for the lack of differences in B2 or B3 between housing conditions. In a cross-sectional design, we may have seen group differences similar to B1 in middle and old age. Overall, our data show that upon first presentation of the task, the NSH group was more likely to employ a random, less efficient exploratory approach early in training (mixed strategy), while the SH group used a more efficient procedural approach (serial strategy) early on.

Different exploratory approaches in B1 likely accounted for the shorter latencies displayed by the SH group. Importantly, there were no group differences in maximum velocity or in time spent freezing in the center at the start of each trial. In addition, we previously reported that this cohort of rats displayed no group differences in locomotor activity or anxiety levels as assessed by the open field maze and blood serum corticosterone levels, respectively (Templer, Wise, & Heimer-McGinn, 2018). As noted in that publication, one limitation is that we did not quantify home cage activity levels. Despite this gap, we did observe that SH rats were more likely to huddle in large groups and rest, while NSH rats more often used the running wheel. This is consistent with research showing increased locomotor activity in isolated rodents (Ago, Takuma, & Matsuda, 2014). Taken together, these data indicate that the shorter latencies in B1 were more likely due to strategy selection than to locomotor differences. Overall, it is clear that acquisition of the standard BM task set, which could be best assessed in the first battery, was different depending on housing conditions. Although both groups reached equal levels of performance in the standard BM, the SH rats reached the GB faster because they employed a more efficient strategy early on.

### Social housing improves flexibility in the variable location BM

The variable BM was a more difficult task because the GB location could never be committed to memory. Perhaps for this reason, or due to interference from the previously tested standard BM, neither of the groups learned the task well upon first presentation in B1. However, upon second presentation in B2, by which point most rats used the more efficient procedural strategy (efficient considering the constantly changing task sets), SH rats reached plateau performance sooner than the NSH rats. Because the GB changed every trial, acquisition of task set required cognitive flexibility in response to changing task demands, especially considering rats had previous experience in the standard BM. In fact, it may be that for the variable BM specifically, our longitudinal design actually enhanced group differences. In a simpler cross-sectional setup, we speculate that group differences may be less evident because there would no longer be a need to adapt to a changing task set. In our design, adaptability across tasks was essential and required (1) employing an efficient strategy and (2) spending less time visiting the previously rewarded location. We explored both of these possibilities and found that, unlike in the standard BM, search strategies could not account for performance advantages in SH rats. The SH group, however, did commit less perseverative errors, and this group difference was correlated with enhanced performance in the second battery. Thus, it is apparent that the SH group had a heightened awareness of and/or flexibility in response to the fact that the GB was not stationary. This is particularly noteworthy because it suggests that the enhancements observed in the SH group originated from cognitive features rather than navigational strategies, which is consistent with results from the first standard BM. This supports the idea that sociality confers cognitive advantages to learning, albeit not spatial in nature.

### Socially housed rats outperform nonsocially housed rats in the reversal BM

In the reversal BM task, SH rats once again showed enhanced cognitive flexibility, this time evidenced by decreased primary latency at the beginning of reversal learning. This effect was slight but significant and was predictably more prominent upon first presentation of the task. In a cross-sectional design we may have seen an even larger effect and perhaps an enhanced effect in old age as well. Unlike the other two task versions, neither strategies nor perseverative errors could account for the group differences in latency. Absence of perseverative errors indicated that spatial memory did not account for the differences in latencies. This is again consistent with the standard and variable location tasks, where procedural, rather than spatial differences accounted for reduced latencies in the SH group. Although the specific mechanisms are not clear, SH rats likely outperformed NSH subjects in the reversal task for the same reason found in all previous tasks: enhanced acquisition of task set. The first time encountering the standard BM task (constant GB location) requires acquisition of task-set in the same way that encountering a changing GB location does, or when the GB suddenly changes from one location to another in reversal learning. In other words, performance advantages by SH rats in the three BM tasks could be functionally equivalent in reflecting enhanced cognitive flexibility. Moving forward, an attentional set-shifting task or 5-choice serial reaction time task would provide further insight into whether sustained social housing improves cognitive flexibility overall.

### Social housing does not affect spatial memory in long-term probes

Finally, in the spatial memory probes performed after standard BM training, no housing differences were observed at any time point. The finding that social enrichment produced latency differences during the training phase but not in the long-term retention of spatial memory is consistent with the effects of EE in previous studies (Bonaccorsi et al., 2013; Harati et al., 2011; Moritz et al., 2014). These studies find that EE enhances spatial learning latency during training, but not spatial memory as assessed by long-term retention probes. Interestingly, these are the same studies mentioned before, which house considerably more subjects per cage in the EE compared to the control groups. This strengthens the idea that it may have been the social and not the physical components of EE that conferred spatial learning benefits in the adult subjects. Finally, the finding that long-term retention of spatial memory was not affected by housing conditions is also consistent with our previous finding that sustained social housing does not influence reference memory in the radial arm maze (Templer, Wise, & Heimer-McGinn, 2018).

### Social housing may enhance overall cognitive flexibility

Overall, careful examination of our results in the first battery (before task sets began to change) suggests that although SH rats did reach the GB faster, their advantage was fueled by faster acquisition of task set, rather than acquisition of spatial cues. That there were no differences in spatial learning and memory is clear, as evidenced by the fact that both groups used the direct strategy increasingly across trials in the standard BM, and performed equally well in long-term memory probes. The key difference in the standard BM was that the SH group immediately implemented a serial strategy at the start of the first battery while the NSH group initially squandered time using a mixed strategy. In the variable BM, where reward locations changed constantly, the SH group was quicker to learn that the originally baited location (from the standard BM) was no longer meaningful. This demonstrated increased flexibility following a change in task set. In the reversal task, the SH group also demonstrated greater adaptability when a stable rewarded location shifted. Finally, a non-spatial simple discrimination task showed a possible trend towards enhanced acquisition in SH rats. Considering these results, the most parsimonious explanation is that social housing enhanced overall cognitive flexibility and acquisition of task set, rather than spatial learning itself. These results are consistent with our previous findings that SH rats display more efficient task set acquisition when assessed for grit/industriousness (Hemmer et al., 2019). This is in line with research showing that play-fighting with peers enhances task switching and overall cognitive flexibility (Pellis, Pellis, & Himmler, 2014). These authors conclude that by play-fighting, rats place themselves in situations of uncertainty and unpredictability with respect to others, which provides an opportunity to improve executive function and become more behaviorally flexible overall. Taken together, these data could indicate that the housing groups made use of different memory systems to acquire the task sets, a possibility which has been previously suggested (Squire, 2004).

### Possible mechanisms underlying social housing benefits

The finding that cognitive flexibility, but not spatial memory, is affected by social enrichment, provides insight into the underlying mechanisms by which sociality confers cognitive benefits. These results are consistent with work from our laboratory showing that SH rats display enhanced working memory compared to NSH rats in old age (Templer, Wise, & Heimer-McGinn, 2018). These cognitive abilities (working memory, reversal learning, flexibility to changing task demands) are considered executive functions and are attributed to the prefrontal cortex (PFC) (Brigman, Graybeal, & Holmes, 2010; Dalley, Cardinal, & Robbins, 2004; Miyake et al., 2000; Remmelink, Smit, Verhage, & Loos, 2016). In contrast, cognitive domains that are considered hippocampus-dependent (Clark, Broadbent, & Squire, 2005), were unaffected by social housing in our longitudinal design. This included spatial learning and spatial long-term memory in the current report and reference memory in previous reports (Templer, Wise, & Heimer-McGinn, 2018). Thus, it is possible that the physiological mechanisms by which social enrichment confers benefits involve areas of the PFC more heavily than those of the hippocampus or other MTL structures. This is consistent with non-longitudinal findings that social isolation impacts reversal learning and intra-dimensional shifts but not spatial memory (Gelfo, 2019; Schrijver, Pallier, Brown, & Wurbel, 2004). Our longitudinal setup, which compares sustained social versus nonsocial housing (with physical enrichment in both), provides an ideal design for studying these processes. In particular, our design boasts high ecological validity because it takes into account previous lifetime experiences and also models a milder nonsocial lifestyle (rather than complete isolation) in the presence of physical activity. For a more detailed discussion on the effects of social enrichment in longitudinal versus cross-sectional designs, see Templer, Wise, and Heimer-McGinn (2018).

### The benefits of social housing go beyond those of EE

Our study shows that the effects of social housing are independent from other forms of enrichment and likely go beyond the benefits of physical enrichment. As outlined in the introduction, an extensive literature review suggests that the effects of EE vary across studies depending on the number of subjects per cage in the enriched versus control conditions. While EE generally confers spatial learning enhancements in aged rats and in disease/injury models (outlined in the introduction) it often has no effects in healthy adult control subjects, (Gortz et al., 2008; Harburger, Lambert, et al., 2007; Harburger, Nzerem, et al., 2007; Jacotte-Simancas et al., 2013; Madhavadas et al., 2017; Peruzzaro et al., 2013; Reichmann et al., 2016). Since the studies that do report spatial learning benefits are those in which the number of rats per cage is significantly larger in EE (10–12 rats) compared to standard conditions (single or pair housing) (Harati et al., 2011; Moritz et al., 2014), it is likely that the cognitive enhancements observed were a consequence of the social component of EE. Notably, while Moritz observed decreased latency in the BM for EE shams compared to standard shams, search strategies were not analyzed. This leaves open the possibility that the difference between these two groups actually reflects task set acquisition rather than a spatial learning enhancement. Taken together, this suggests that while the cognitive benefits of EE are apparent in middle to old age and in disease models, sociality has the added benefit of enhancing cognition earlier in healthy adults. Since all subjects were environmentally enriched and handled extensively in our experimental design, it is clear that social housing exerts additional benefits beyond those of EE. For human populations, this implies that sociality could be beneficial even for healthy adults who lead otherwise active lifestyles.

### Concluding Statements

Understanding how social exposure influences cognitive capacity is fundamental to many fields, including neuropsychiatric diseases, aging, and recovery from neurological injury. Overall, we conclude that sustained social housing enhances cognitive flexibility at all ages as evidenced by superior task set acquisition (standard BM), adaptability to a new task set (variable BM), and enhanced performance in a reversal task (reversal BM). Consistent with our previous study (Templer, Wise, & Heimer-McGinn, 2018), social housing appeared to affect PFC-mediated executive functions, rather than hippocampus-dependent behaviors like retention of long-term spatial memory. Since the benefits of sociality went beyond those of physical enrichment at all time-points, the implications of our work may be relevant to adults of all ages leading otherwise enriched lifestyles. Importantly, our studies provide proof of concept for the use of rodent studies with housing condition as an independent variable. Future studies should include female rodents so as to increase ecological validity. Such a model can be used in future studies to isolate the neural mechanisms underlying preserved cognitive functions associated with social enrichment. This knowledge will be instrumental for the development of treatments and preventive care for cognitive dysfunction in adults.

#### Open Practices Statement

Neither of the experiments reported in this article was formally preregistered. Neither the data nor the materials have been made available on a permanent third-party archive; requests for the data or materials can be sent via email to the lead author at vtempler@providence.edu.

## Supplementary Material

13420_2020_418_MOESM1_ESM

## Figures and Tables

**Table 1:** Task layout by battery, testing day, and correct goal-box location

Battery	Task	PND	Correct GB
Battery 1	Standard BM	142–148	17
	Probe	156,157,158,159	17
	Variable BM	163–168	11,3,1,17,15,17,3,7

Battery 2	Standard BM	427–431	7
	Probe	436,443	7
	Variable BM	503–506	14,10,5,18,9,3,12,2
	Reversal BM	510–513	10, 1

Battery 3	Standard BM	671–675	4
	Probe	678, 680, 682	4
	Variable BM	684–688	14,10,5,18,9,3,12,2
	Reversal BM	719–722	1, 10

Abbreviations-BM: Barnes maze; PND: post-natal day; GB: goal box
