# Supplementary material for: Social housing enhances acquisition of task set independently of environmental enrichment: A longitudinal study in the Barnes maze
Source: Learn Behav. Author manuscript; Available in PMC 2021 Sep 1. (PMC7415481; doi:10.3758/s13420-020-00418-5)
Supplement: 13420_2020_418_MOESM1_ESM [file NIHMS1559365-supplement-13420_2020_418_MOESM1_ESM.docx]

Supplemental materials


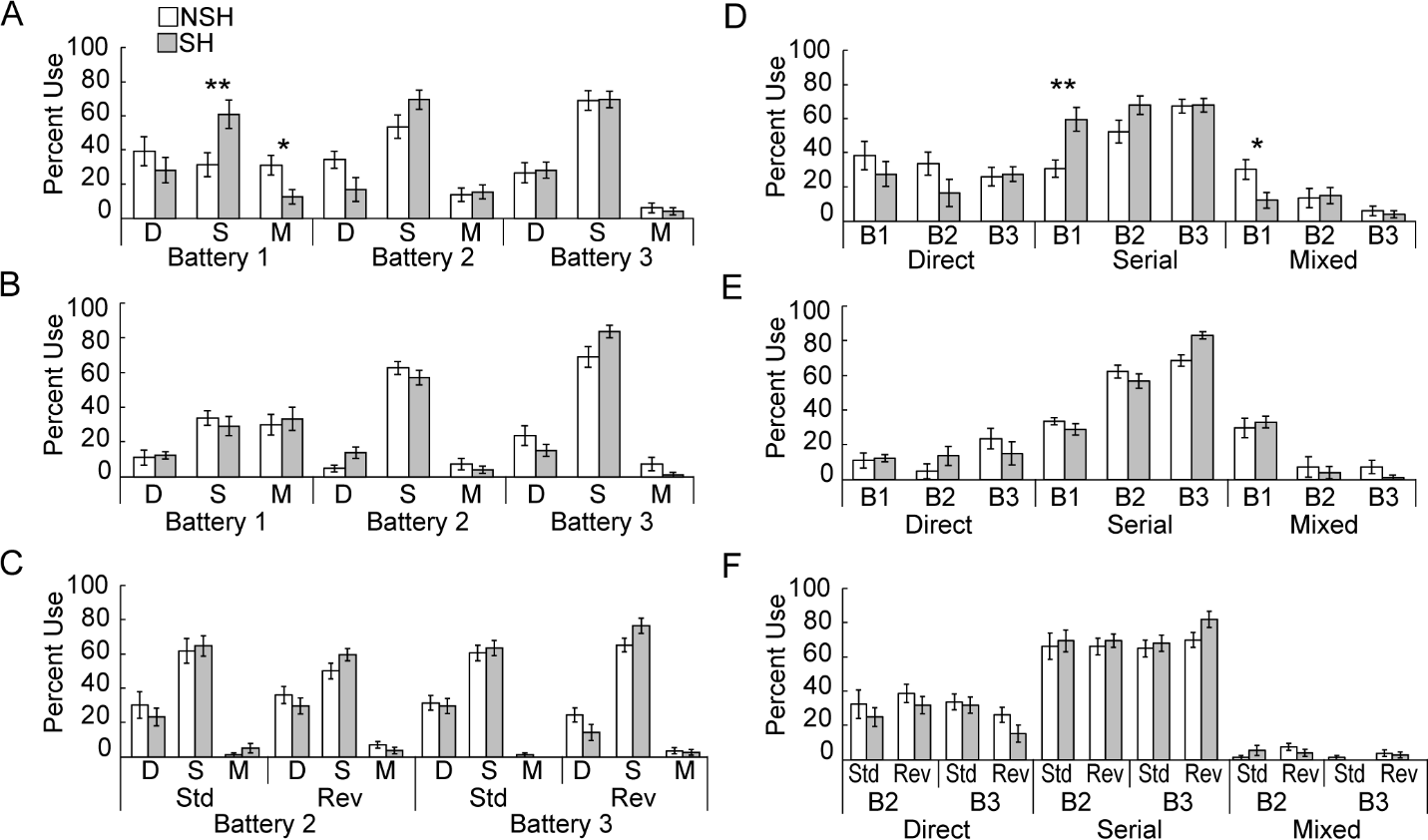


**Fig. S1** Mean percentage use of strategies in Batteries 1, 2, and 3 (B1, B2, and B3, respectively) for the standard Barnes maze (**a, d**), variable location Barnes maze (**b, e**), and reversal Barnes maze (**c, f**). The data in **a–c** are organized by battery, while in **d–f** they are organized by strategy. Abbreviations: Std = standard training; Rev = reversal learning. Strategies were categorized as either direct (D), serial (S), or mixed (M). Error bars represent standard error of the mean. Significance for one-way ANOVAs: * indicates *p* < .05, ** indicates *p* < .01





**Fig. S2** Use of strategies across trials. For each trial, data points represent the percent of subjects per group that used a given strategy. Panels on the left side depict the nonsocial group while panels on the right side depict the social group. For each group, strategies used in the standard (top), variable location (middle), and reversal (bottom) tasks are shown. The top panel is presented in the main article, but is included here for ease of comparison. Abbreviations: BM = Barnes maze; B2 = Battery 2; B3 = Battery 3; Rev = reversal phase; Std = standard phase of the reversal task

**Simple discrimination task**

**Method**

**Procedure.** The simple discrimination task required subjects to use a perceptual dimension, either olfactory or sensory, to find a food reward. Rats in each housing group were randomly assigned to either odor or medium discrimination and tested in a square arena (30 × 15 in). A wooden floor was lined with white contact paper, and the walls were made from large Duplo blocks stacked to a height of 9 inches. An adjacent starting chamber (11 × 15 in) made of the same materials was accessible by guillotine door. After 30 seconds in the starting chamber, subjects were allowed into the testing area and a trial began. During each trial, two terra cotta pots (4 × 4.5 in) with distinct odors or mediums were presented and only one odor or medium indicated the location of the food reward (6 whole Fruit Loop pieces). Both pots contained pulverized cereal mixed in with the media to control for smell. The trial ended when the rat either retrieved one cereal piece from the correct pot, or began to dig in the incorrect pot. Each rat was presented with a minimum of six trials and tested until they obtained six correct pot choices in a row. For the odor-relevant cohort, both pots were filled with bedding, and either lemon or cinnamon scents indicated the location of the reward. For the medium-relevant cohort, no odor was applied, and the reward was buried under either pipe cleaners or crinkled paper strips. The correct scent or medium was counterbalanced across rats and the location of the rewarded pot (left or right) was counterbalanced across trials for each rat. Pots were 4 inches away from each other and 7 inches away from the back wall.

**Statistical analysis.** Trials to criterion (TTC) was defined as the number of trials it took each subject to correctly locate the reward on six consecutive trials. Since TTC values could not be less than 6, and since 6 was the most common TTC score, the data were not normally distributed, as shown by Shapiro–Wilk tests for the NSH (*p* = .022) and SH (*p* = .000) groups. Because both groups had similar nonnormal distributions, the nonparametric Mann–Whitney *U* test was applied and interpreted as a comparison of means.

**Results**

Mean TTC ± *SEM* was 6.78 ± 1.56 for the NSH and 9.20 ± 4.10 for the SH (see Fig S3a). A nonparametric Levene’s test showed unequal variances (*p* = .023) but despite the considerable mean difference, the groups were not statistically different (*U* = 26.50, *p* = .092). Since the SH group had mostly perfect scores (i.e., 6) while the NSH group did not, we also analyzed the data using chi-squares. Once again, although the SH group had a higher proportion of perfect TTC scores than the NSH group (see Fig. S3b), this difference was not statistically significant, χ^2^(2 *n* = 19) = 2.773, *p* = .096.

**

Fig. S3** Performance on a simple discrimination task. **a** Trials to criterion (TTC) by group. **b** Chi-square graph comparing the number of subjects with a perfect score (i.e., TTC equals 6) to those that made at least one incorrect choice (i.e., TTC greater than 6). Error bars represent standard error of the mean (*SEM*)

**Discussion**

The SH group had consistently perfect scores with a low standard error while the NSH group had a higher mean score and standard error. Although no statistical significance was observed, the *p* values obtained could be interpreted as loosely “trending.” More importantly, since the SH group performed at near ceiling levels, it is possible that the simplicity of the task precluded a nuanced comparison between groups. Indeed, it has been reported that statistical analysis of plateaued data can be inadequate ([van den Berg & Ma, 2014](#_ENREF_44)). Thus, further testing is required to conclusively report whether social housing confers learning benefits in a nonspatial task.

**Reference**

van den Berg, R., & Ma, W. J. (2014). “Plateau”-related summary statistics are uninformative for comparing working memory models. *Attention, Perception, & Psychophysics, 76*(7), 2117–2135. doi:10.3758/s13414-013-0618-7
